# Dapsone Ameliorates Isoproterenol-Induced Myocardial Infarction *via* Nrf2/ HO-1; TLR4/ TNF-α Signaling Pathways and the Suppression of Oxidative Stress, Inflammation, and Apoptosis in Rats

**DOI:** 10.3389/fphar.2021.669679

**Published:** 2021-05-19

**Authors:** Walaa Yehia Abdelzaher, Sabreen Mahmoud Ahmed, Nermeen N. Welson, Khalaf F. Alsharif, Gaber El-Saber Batiha, Dina A. Aly Labib

**Affiliations:** ^1^ Department of Pharmacology, Faculty of Medicine, Minia University, Minia, Egypt; ^2^ Depatment of Human Anatomy and Embryology, Faculty of Medicine, Minia University, Delegated to Deraya University, Minia, Egypt; ^3^ Department of Forensic Medicine and Clinical Toxicology, Faculty of Medicine, Beni-Suef University, Beni-Suef, Egypt; ^4^ Department of Clinical Laboratory Sciences, College of Applied Medical Sciences, Taif University, Taif, Saudi Arabia; ^5^ Department of Pharmacology and Therapeutics, Faculty of Veterinary Medicine, Damanhour University, Damanhour, Egypt; ^6^ Department of Pharmacology, Faculty of Medicine, Cairo University, Giza, Egypt

**Keywords:** dapsone, myocardial infarction, Nrf2, HO-1, TLR4

## Abstract

Myocardial infarction (MI) is a critical condition that can happen with high doses or rapid termination of beta blockers therapy. The study aimed to evaluate the potential anti-toxic value of DAP against isoproterenol (ISO) - induced MI. Twenty-eight male Wistar rats were used for the study. The rodents were assigned to four groups (*n* = 7) and the treatments were given for 12 days as follows; Group 1 (control): were administrated normal saline, Group 2 (DAP control): were administrated DAP (10 mg/kg/day IP), Group 3 (ISO group): were administrated ISO (100 mg/kg, IP on the 11th and 12th days of the experiment), and Group 4 (DAP + ISO): co-treated with DAP plus ISO. The measured parameters were cardiac malondialdehyde (MDA), reduced glutathione (GSH), total nitrite/nitrate (NOx), catalase (CAT), serum cardiac biomarkers; CK-MB, ALT, LDH, and ALK-PH. Also, interleukin-1β (IL-1β), tumor necrosis factor-alpha (TNF-α), Nuclear factor (erythroid-derived 2)-like 2 (Nrf2), heme oxygenase-1 (HO-1), toll-like receptor 4 (TLR4), caspase-3 activity, and hepatic BAX and Bcl-2 were also assessed. Also, histological examination and vimentin immuno-expressions were studied. ISO group exhibited MI as evidenced by the elevation in serum cardiac biomarkers, MDA, NOx, IL-1β, TNF-α, and caspase-3 together with the reduction in GSH, Nrf2, HO-1 levels, and a faint vimentin immuno-reaction. Histological alterations revealing distorted cardiomyocytes; vacuolation, edema, pyknosis, and fragmentation were also noticed. DAP significantly ameliorated all the examined toxicity indicators. DAP revealed efficient ameliorative actions against ISO-caused MI by marked reduction in myocardial infarct size and suppressed oxidative stress, inflammation, and apoptosis via the up-regulation of the Nrf2/HO-1; TLR4/TNF-α signaling pathways.

## Introduction

Myocardial infarction (MI) is a devastating event, mainly if reperfusion does not occur (Torina et al., 2015). MI results from the imbalance between blood supply and demand. Many mechanisms are implicated in the pathogenesis of MI but the suggested roles of these mechanisms are still unclear. The disrupted myocardial oxidants\antioxidants balance, the triggered inflammation, and the decreased cell viability influence cardiac function (Shahat et al., 2016; Khalaf et al., 2020). Apoptosis and necrosis are essential in the pathogenesis of MI (Garg et al., 2016). Myocardial inflammation, calcium overload, coronary spasm, and death of the myocytes result from the over-stimulation of myocardial β-adrenergic receptors. This leads to an increase in the cAMP concentration causing more stimulation of protein kinase A and phosphorylation of L-type calcium channels (Euteneuer et al., 2012).

Isoproterenol (ISO) is a synthetic nonselective β-adrenergic agonist. Acting on the β1 adrenergic receptors in the heart, high doses of ISO can lead to calcium overload, myocardial oxidative stress, coronary hypotension, and energy depletion. Therefore, searching pharmacological methods to improve isoproterenol-induced acute cardiac toxicity is important for stopping the initiation and deterioration of MI (Bhandari et al., 2008; Garg et al., 2016).

Nuclear factor (erythroid-derived 2) - like 2 (Nrf2) plays a major role in the anti-oxidant, anti-inflammatory responses. Nrf2 has a cardio-protective activity as it protects against maladaptive remodeling and decreased cardiac function (Zhou et al., 2014).

Heme oxygenase-1 (HO-1), the inducible type of HO, has a cytoprotective effect mainly in inflammatory conditions (Zhang et al., 2015). Through its down-regulation of the inflammatory cytokines, HO-1 can protect the cells against the inflammatory damage (Megías et al., 2009; Zhang et al., 2015). HO-1 is induced by oxidative or nitrosative stress, cytokines, and other mediators produced during inflammatory processes, likely as part of a defense system in cells exposed to stress to provide negative feedback for cell activation and the production of mediators, which could modulate the inflammatory response (Alcaraz et al., 2003).

Oxidative stress up-regulated pro-inflammatory cytokine synthesis. Toll-like receptor 4 (TLR4), a member of the transmembrane recognition proteins family, is essential for the stimulation of inflammation noticed in experimental ischemia/reperfusion (I/R). Activation of TLR4 ultimately stimulates the pro-inflammatory cytokines release e.g., tumor necrosis factor-α (TNF-α) and interleukins-1β (IL-1β) (Ibrahim et al., 2021).

Dapsone (DAP) is a sulfonamide antimicrobial agent used in the treatment of leprosy in combination with rifampicin and clofazimine. DAP inhibits the dihydrofolic acid synthesis in bacteria leading to inhibition of bacterial growth (Wozel and Blasum, 2014). DAP has a dual function; it has anti-inflammatory effects plus antimicrobial activity. It reduces the synthesis of the pro-inflammatory messengers and TNF-α production (Sheibani et al., 2020).

DAP improved functional deficit and diminished brain damage after transient cerebral ischemia and reperfusion in rats (Diaz-Ruiz et al., 2016). DAP also had remarkable anti-convulsive, neuroprotective and antioxidant effects. Regarding its anti-oxidant effects, it decreased extracellular reactive oxygen species (ROS) and suppressed the synthesis of superoxide anion by interacting with the membrane-bound NADPH oxidase (Rios et al., 2015; Zhan et al., 2018).

To date, the efficacy of DAP on MI has not been investigated or audited. Consequently, the objective of this study is to examine the possible ameliorative function of DAP against ISO-induced MI in rats through the attenuation of inflammatory, oxidative stress, and apoptotic responses.

## Materials and Methods

### Ethics

Animal handling, medications, and scarification were carried out following the guidelines for the care of experimental animals and approved by the Institutional Ethical Committee, Medicine Faculty, Minia University, Egypt according to the NIH Guide for taking care and use of laboratory animals (Approval No. 709:12/2020).

### Chemicals

ISO was bought from Sigma-Aldrich Co. (St. Louis, MO, United States) and DAP was obtained from GlaxoSmithKline Pharmaceuticals Ltd. Reduced glutathione (GSH) and catalase (CAT) kits were purchased from Biodiagnostic, Giza, Egypt. Alanine transaminase (ALT) kit was from Spectrum Diagnostic, Cairo, Egypt. Interleukin (IL)-1β ELISA kit (Nanjing Jiancheng Bioengineering, Nanjing, China), Nrf2 ELISA kits (MyBioSource, Inc., San Diego, United States), Tumor necrosis factor (TNF)-α ELISA kit (IDlabsT-Minc. Biotechnology, Canada), and TLR4 ELISA kits (Sigma-Aldrich Co. (St. Louis, MO, United States) were used. Serum creatinine kinase-MB (CK-MB), lactate dehydrogenase (LDH), and alkaline phosphatase (ALK-PH) were purchased using commercial kits (MyBio Source Co.). Caspase-3 was quantified by ELISA kit (Cusabio, United States). Heme oxygenase-1 (HO-1) was obtained from Assay Designs, Ann Arbor, MI, United States. Other chemicals were of analytical grade and were bought from commercial sources.

### Animals and Experimental Design

Twenty-eight male albino Wistar rats weighing 180–210 g were procured from the National Center of Research, El-Giza, Egypt. After two weeks of acclimatization to the environmental conditions (12 h lighting cycle, 25 ± 2°C temperature, 45 ± 5% humidity, and free access to water and standard chow from El-Nile Company, Egypt), the rodents were assorted into 4 groups (7 rats/group). Group 1 (control): received dimethylsulphoxide (DMSO) for 12 days IP as a vehicle for DAP and saline as a vehicle for ISO in the 11th and 12th days IP. Group 2 (DAP): treated with dapsone (DAP) 10 mg/kg/day IP for 12 days (12). Group 3 (ISO): treated with saline orally for 12 days and isoproterenol (ISO) was injected (100 mg/kg/day) in the 11th and 12th days IP (3). Group 4 (ISO + DAP): received DAP (10 mg/kg/day for 12 days) + ISO in the regimen as group3.

### Assessment of Myocardial Infarction

The isoproterenol-induced myocardial infarction was assessed by measuring myocardial infarct size using 2,3,5-triphenyltetrazolium chloride (TTC) staining method (Fishbein et al., 1981; Babbar et al., 2013).

### Blood and Tissue Sampling

To terminate the experiment, the rodents were sacrificed after overnight fasting. They were anesthetized with an IP injection of urethane (25% in a dose of 1.6 gm/kg). Blood was obtained from the abdominal aorta, placed in heparinized syringes, left to coagulate at room temperature, centrifuged at 5,000 rpm for 15 min (JanetzkiT30 centrifuge, Germany), and then sera were frozen at -80°C for further analysis. The hearts were excised, washed from the blood by saline, and divided. Samples from the base of the left ventricle were kept in 10% formal-saline for histopathological and immunohistochemical studies. Other cardiac specimens were homogenized in ice-cold phosphate buffer (0.01 M, pH 7.4; 20% w/v), 10 mM pH (7.4). The ratio of tissue weight to homogenization buffer was 1:5. Then tissue centrifuged for 15 min at 5,000 rpm, and the supernatant was frozen at -80°C for further biochemical examination.

### Biochemical Analysis

#### Quantification of Oxidative Stress Parameters in Cardiac Tissue

Malondialdehyde (MDA) level, the index of lipid peroxidation, was determined following the method of Buege and Aust, 1978. Total nitrite/nitrate (NOx), the stable oxidation end products of nitric oxide, was used as an indicator of the nitric oxide level and was assessed by the reduction of nitrate into nitrite using activated cadmium granules followed by color development with Griess reagent in an acidic medium (Sastry et al., 2002). GSH and CAT were measured using colorimetric kits and following the manufacturer’s instructions.

#### Determination of Cardiac Biomarkers

The serum levels of CK-MB, ALT, LDH, and ALK-PH were quantified using commercial kits and following the manufacturer’s instructions.

#### Measurement of Cardiac TLR4, TNF-α, IL-1β, HO-1, Nrf-2, and Caspase-3

Cardiac TLR4, TNF-α, IL-1β, HO-1, Nrf-2, and caspase-3 were determined using their ELISA kits and following the manufacturer’s instructions.

### Real-Time Reverse-Transcription Polymerase Chain Reaction (RT-PCR) of Bax and Bcl2 Gene Expression

RT-PCR for the relative quantification of the apoptotic Bax and the anti-apoptotic Bcl2 genes was done in liver tissue. Total RNA was extracted from the homogenized hepatic specimen using Ribozol RNA extraction reagent (Amresco, Solon, United States) following the manufacturer’s instructions. cDNAs were synthesized using a RevertAid™ First Strand cDNA Synthesis kit (Fermentas, Life Sciences). cDNA was reversely transcribed from 5 μg of mRNA in transcription buffer, 200 U M-MuLV Reverse transcriptase, and 20 U RNase inhibitor at 42°C for 60 min followed by immediate cooling on ice. RT-PCR was performed with 50 ng cDNA per reaction using 25 μl of SYBR Green QPCR Mix (Solis BioDyne) containing 20 μm of specific primers in the Real-Time PCR Detection System. The SYBR green data were analyzed with a relative quantification to GAPDH (glyceraldehyde-3-phosphate dehydrogenase) as a reference gene. The sets of primers used were as follows:

Bax forward, 5′-GGA​GAC​ACC​TGA​GCT​GAC​CT-3′, and reverse, 5′-CTC​AGC​CCA​TCT​TCT​TCC​AG-3 (Kumar et al., 2015); Bcl2 forward, 5′-TATATGGCCCCAGC ATGCGA-3′, and reverse, 5′-GGGCAGGTTTGTCG ACCTCA-3 (Jafari et al., 2015).

GAPDH forward primers are as follows: 5′ GTC​GGT​GTG​AAC​GGA​TTT​G3′ and reverse 5′ CTT​GCC​GTG​GGT​AGA​GTC​AT3′ (Wimmer et al., 2018).

The relative expression level of each gene was calculated using formula 2 (-ΔΔCt) according to VanGuilder et al. (2008). They were scaled relative to controls where control samples were set at a value of 1. Thus, results for all experimental samples were graphed as relative expression compared with the control.

### Histopathological Analysis

#### Histological Study

Samples of the base of the left ventricle were kept in 10% formal-saline and then paraffin sections (5–7 μm thickness) were placed on glass slides for hematoxylin and eosin (H&E) stain and Masson’s trichrome (MTC) staining for demonstration of collagen fibers (Kamel et al., 2020).

#### Immunohistochemical Study

Other sections were dewaxed in xylene, rehydrated and 3% hydrogen peroxide was added for blocking of peroxidase action. Sections were incubated over night at 4°C with monoclonal anti-vimentin anti body for demonstration of cell regeneration (Lapvision Inc., Fremont, CA, United States). Sections were washed with PBS, incubated with IgG and then with strept avidin-peroxidase. Sections were then washed with PBS and diaminobenzidine (DAB) was added for 5 min. Finally, the sections were stained with MayER’s hematoxylin (Helal et al., 2010).

### Morphometric Analysis

Measurements were carried out in five non-overlapping fields from five various sections of five various rats in each group at × 100 magnifications using the image analyzer (Leica Imaging System, Germany) (Aziz et al., 2020) to measure:

1) Area% of the collagen fibers in MTC-stained sections.
2) Area% of the immunopositive expression of vimentin.

The histopathological changes were scored from 0 to three in relation to their severity; where 0 denotes no pathologic finding, and one, two, and three denote pathologic results of < 33, 33–66, and > 66% of the tissue respectively (Welson et al., 2020).

### Statistical Analysis

All results were demonstrated as means ± standard error of the mean (SEM). One-way analysis of variance (ANOVA) and the Tukey–Kramar post-analysis test were performed to analyze the values. Less than 0.05 *p*-value was assigned for significance. Graph Pad Prism was used for the statistical analysis (version 5.01 for Windows, Graph pad Software, San Diego California United States, and www.graphpad.com).

## Results

### Effect of DAP on Myocardial Infarct Size

**FIGURE 1:**
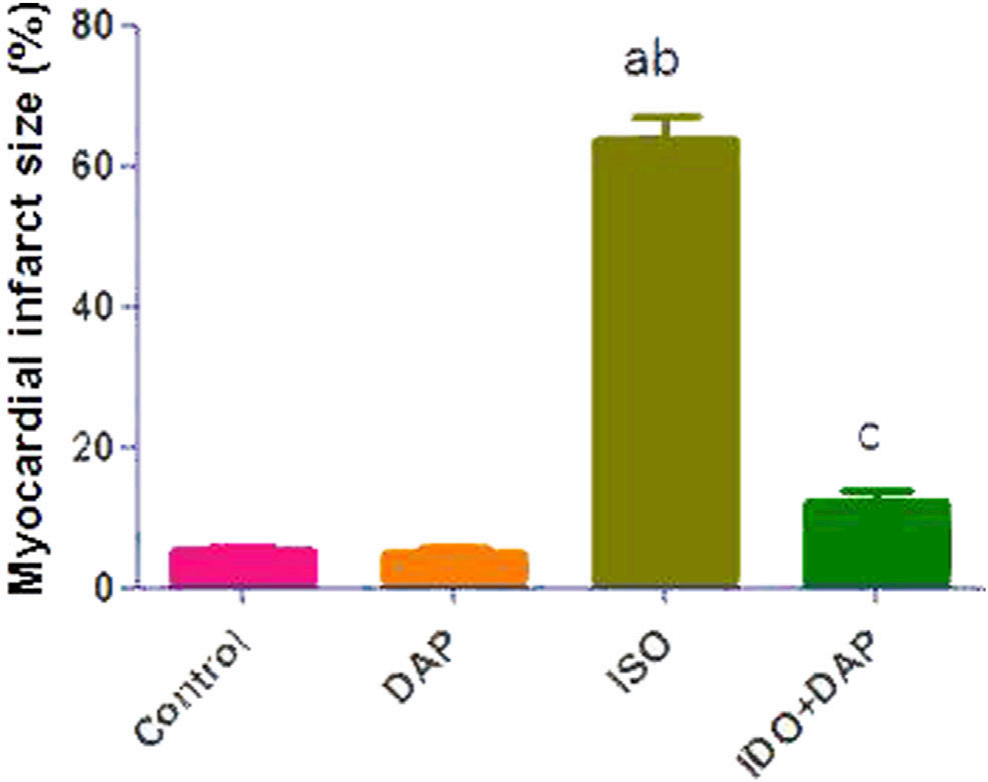
Effect of DAP on myocardial infarct size. Data show the mean ± SEM (*n* = 7). DAP: dapsone; ISO: isoproterenol. ^a^Significant (*p* < 0.05) variance from the control group. ^b^Significant (*p* < 0.05) variance from DAP group. ^c^Significant (*p* < 0.05) variance from ISO group.

A significant increase in myocardial infarct size was noted in ISO group as compared to control and DAP groups (12.7 fold). Meanwhile, rats treated with ISO + DAP significantly prevented ISO-induced high myocardial infarct size (0.19 fold) (Figure 1).

### Effect of DAP on Oxidative Stress Markers in MI in Rats

Comparable to the control and DAP groups, there was a significant increase (*p* < 0.05) in cardiac MDA (2 folds) and NOx (2.5 folds) with significant decrease (*p* < 0.05) in cardiac GSH (0.31 fold) and CAT (0.45 fold) in the ISO group as shown in table 1. However, rats treated with ISO + DAP demonstrated a significant enhancement (*p* < 0.05) of cardiac oxidative stress indicators when compared to the ISO group.

**TABLE 1:** Influence of DAP on oxidative stress markers in MI in rats.

Groups	Cardiac MDA (nmol/g tissue)	Cardiac CAT (U/mg tissue)	Cardiac GSH (mg/g tissue)	Cardiac NOx (nmol/g tissue)
Control	36.97 ± 3.39	51.16 ± 3.39	2.59 ± 0.24	15.58 ± 0.86
DAP	40.67 ± 3.56	50.26 ± 3.12	2.64 ± 0.25	17.85 ± 0.94
ISO	77.74 ± 2.73^ab^	23.11 ± 1.87^ab^	0.83 ± 0.05^ab^	40.65 ± 2.68^ab^
ISO + DAP	44.12 ± 2.47^c^	46.87 ± 2.61^c^	2.09 ± 0.15^c^	20.46 ± 0.98^c^

Data show the mean ± SEM (*n* = 7).

DAP: dapsone; ISO: isoproterenol; GSH: reduced glutathione; MDA: malondialdehyde; CAT: catalase; NOx: total nitrite/nitrate.

a Significant (*p* < 0.05) variance from the control group.

b Significant (*p* < 0.05) variance from DAP group.

c Significant (*p* < 0.05) variance from ISO group.

### Effect of DAP on Cardiac Biomarkers in MI in Rats

Serum levels of CK-MB, ALT, LDH, and ALK-PH were significantly elevated (2.5–3 folds) (*p* < 0.05) in the ISO group in comparison with the control and DAP groups. Rats treated with ISO + DAP displayed a significant reduction (2–2.8 folds) (*p* < 0.05) in cardiac enzymes relative to the ISO group (Table 2).

**TABLE 2:** Influence of DAP on cardiac enzymes in MI in rats.

Groups	CK-MB (U/L)	LDH (U/L)	ALT (U/L)	ALK-PH (U/L)
Control	28.57 ± 2.40	122.10 ± 5.68	23.54 ± 2.11	25.80 ± 2.17
DAP	29.32 ± 2.51	134.60 ± 5.48	24.98 ± 1.86	29.65 ± 2.42
ISO	72.25 ± 3.14^ab^	323.30 ± 2.69^ab^	79.14 ± 2.68^ab^	70.94 ± 2.76^ab^
ISO + DAP	35.69 ± 2.56^c^	143.20 ± 3.49^c^	28.23 ± 2.14^c^	35.00 ± 3.06^c^

Data show the mean ± SEM (*n* = 7).

DAP: dapsone; ISO: isoproterenol; CK-MB: serum creatinine kinase-MB; LDH: lactate dehydrogenase; ALK-PH: alkaline phosphatase; ALT: alanine transaminase.

a Significant (*p* < 0.05) variance from the control group.

b Significant (*p* < 0.05) variance from DAP group.

c Significant (*p* < 0.05) variance from ISO group.

### Effect of DAP on Cardiac TLR4, TNF-α, IL-1β, HO-1, Nrf-2, and Caspase-3 in MI in Rats

Cardiac levels of TLR4, TNF-α, IL-1β, and Caspase-3 were significantly elevated (2.5, 2.4, 1.9, and 3.2 folds respectively) (*p* < 0.05) with significant decrease (*p* < 0.05) of Nrf-2 and HO-1 (0.6 and 0.4 fold respectively) in the ISO group in comparison with control and DAP groups. Rats treated with ISO + DAP showed significant decrease (*p* < 0.05) in cardiac levels of TLR4, TNF-α, IL-1β, and caspase-3 (0.4, 0.5, 0.6, and 0.4 fold respectively) with significant increase (*p* < 0.05) of Nrf-2 and HO-1 (1.6 and 2.1 folds respectively) when compared to ISO group (Table 3).

**TABLE 3:** Effect of DAP on cardiac TLR4, TNF-α, IL-1β, HO-1, Nrf-2, caspase-3.

Groups	Cardiac TLR4 (ng/g tissue)	Cardiac TNF-α (pg/g tissue)	Cardiac IL-1β (pg/g tissue)	Cardiac HO-1 (pg/g tissue)	Cardiac Nrf-2 (pg/g tissue)	Cardiac Caspase-3 (ng/g tissue)
Control	0.99 ± 0.09	29.65 ± 2.02	45.91 ± 2.79	65.23 ± 3.04	81.47 ± 2.93	10.94 ± 0.92
DAP	1.01 ± 0.08	32.35 ± 1.72	50.85 ± 2.75	64.35 ± 2.83	85.39 ± 4.45	11.67 ± 1.03
ISO	2.49 ± 0.17^ab^	71.31 ± 2.67^ab^	85.61 ± 2.91^ab^	26.82 ± 1.64^ab^	54.0 ± 4.98^ab^	35.73 ± 3.16^ab^
ISO + DAP	1.18 ± 0.10^c^	37.06 ± 2.24^c^	52.11 ± 2.33^c^	58.08 ± 2.55^c^	86.79 ± 3.15^c^	14.73 ± 1.34^c^

Data show the mean ± SEM (*n* = 7).

DAP: dapsone; ISO: isoproterenol; TLR4: toll like receptor 4; IL-1β:interleukin-1beta; TNF-α:tumor necrosis factor-alpha; HO-1: heme oxygenase-1; Nrf2:Nuclear factor (erythroid-derived 2)-like 2.

a Significant (*p* < 0.05) variance from the control group.

b Significant (*p* < 0.05) variance from DAP group.

c Significant (*p* < 0.05) variance from ISO group.

### Effect of DAP on Hepatic BAX and Bcl-2 in MI in Rats

**FIGURE 2:**
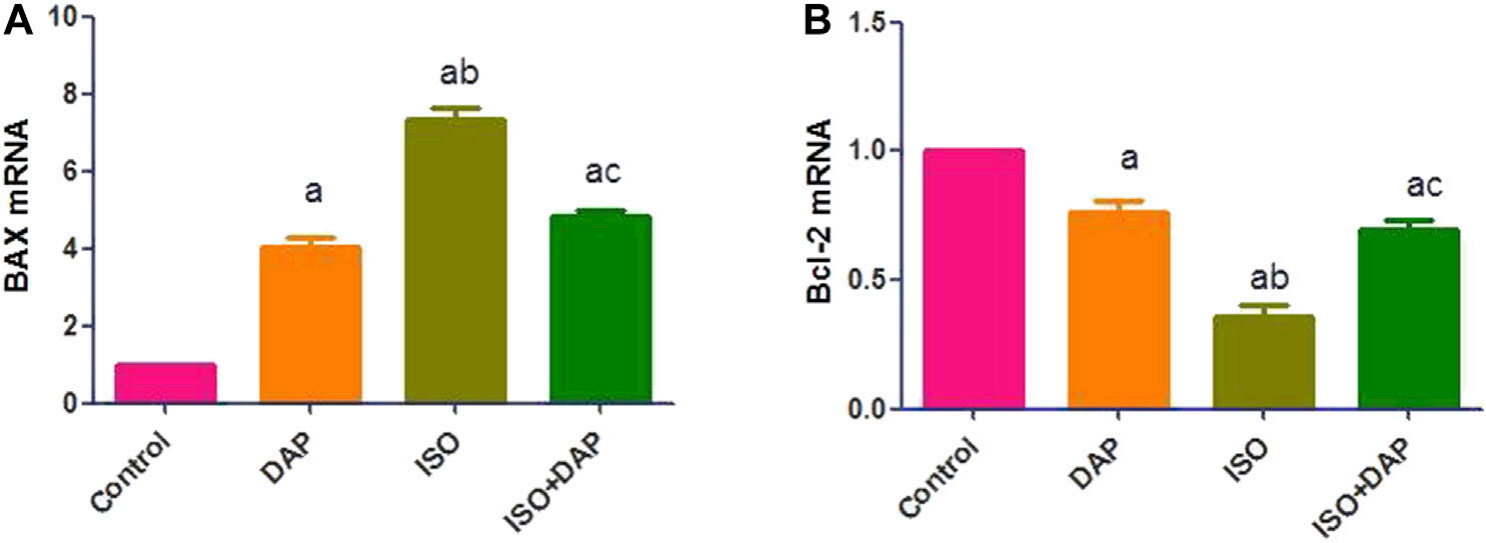
Effect of DAP on hepatic BAX **(A)** and Bcl-2 mRNA **(B)** in MI in rats. Data show the mean ± SEM (*n* = 7). DAP: dapsone; ISO: isoproterenol. ^a^Significant (*p* < 0.05) variance from the control group. ^b^Significant (*p* < 0.05) variance from DAP group. ^c^Significant (*p* < 0.05) variance from ISO group.

ISO significantly (*p* < 0.05) increased the hepatic Bax (7 folds) and decreased the Bcl2 (0.3 fold) mRNA levels in comparison with the control group. Pretreatment with DAP significantly (*p* < 0.05) decreased Bax (0.6 fold) and increased Bcl2 (2 folds) mRNA in comparison with the ISO group (Figure 2).

### Histological Results

#### H and E-Stained Sections

**FIGURE 3:**
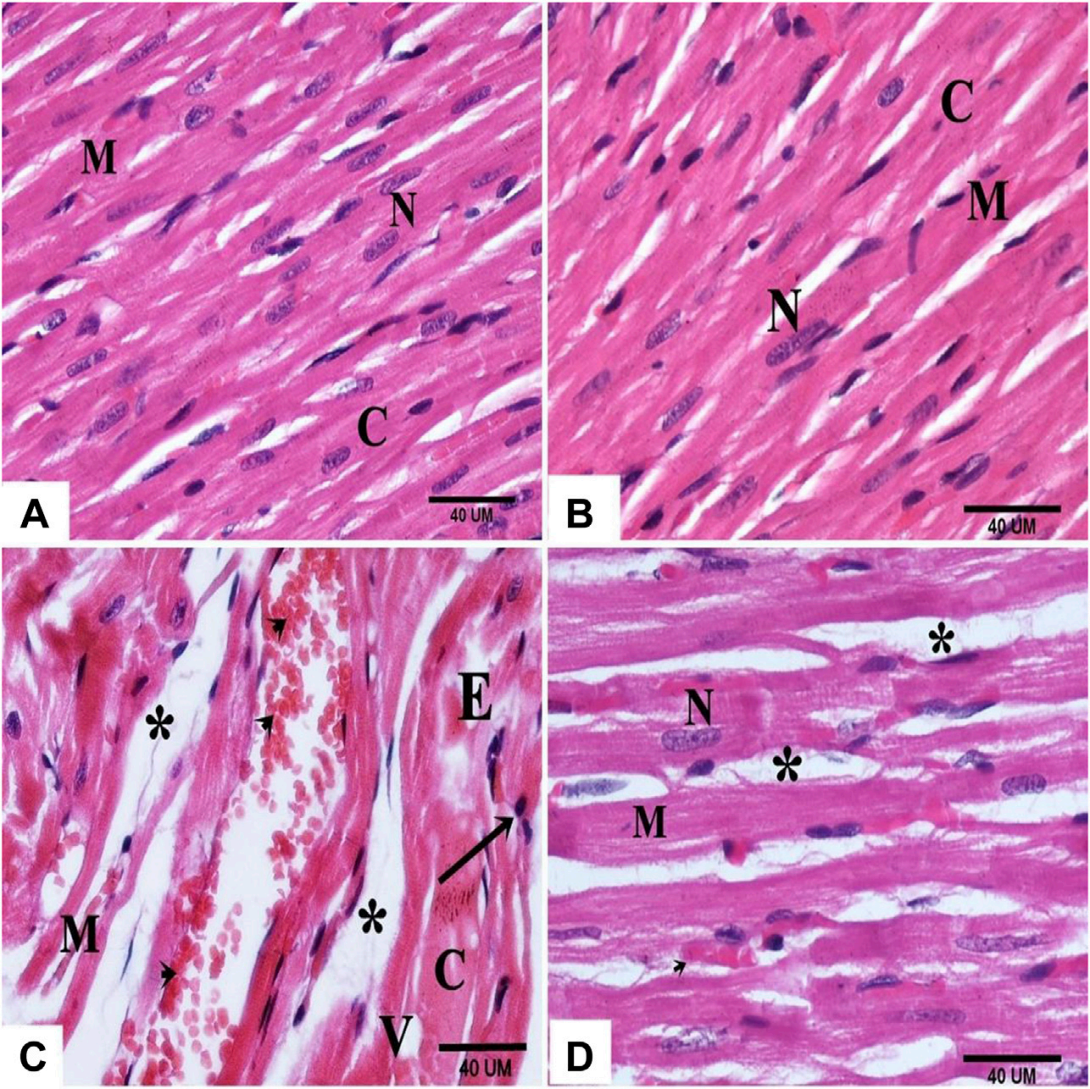
photomicrographs of sections in the rat myocardium **(A and B)** From group (I) and group (II) respectively show intact cardiac muscle fibers (M), central nucleus (N) and acidophilic cytoplasm **(C)**. Group (III) shows structural changes; fragmented (M) and separated (*) cardiac muscle fibers, vacuolation (V), edema (E), pale cytoplasm (C), pyknotic nuclei (arrow) and extravasated RBCʾs (arrow head). **(D)** Group (IV) shows normal myocardial structure except some cardiac muscle fibers (M) with central nucleus (N) and wide spaces (*).

Histological examination of the myocardial sections of the control and DAP- treated rodents displayed normal myocytes with acidophilic sarcoplasm and centrally located nuclei (Figures 3A,B). In the cardiac sections of the ISO group, the myocardial histology was disturbed; cardiomyocytes appeared paler with faint sarcoplasm, vacuolation, edema, and pyknotic nuclei. Many cells appeared separated and fragmented with areas of extravasated RBCs in-between (Figure 3C).

On the other hand, the cardiac sections of the ISO + DAP group revealed almost normal myocardial structure except in limited regions with wide spaces between the cardiac fibers (Figure 3D).

#### Masson’ Trichrome-Stained Sections

**FIGURE 4:**
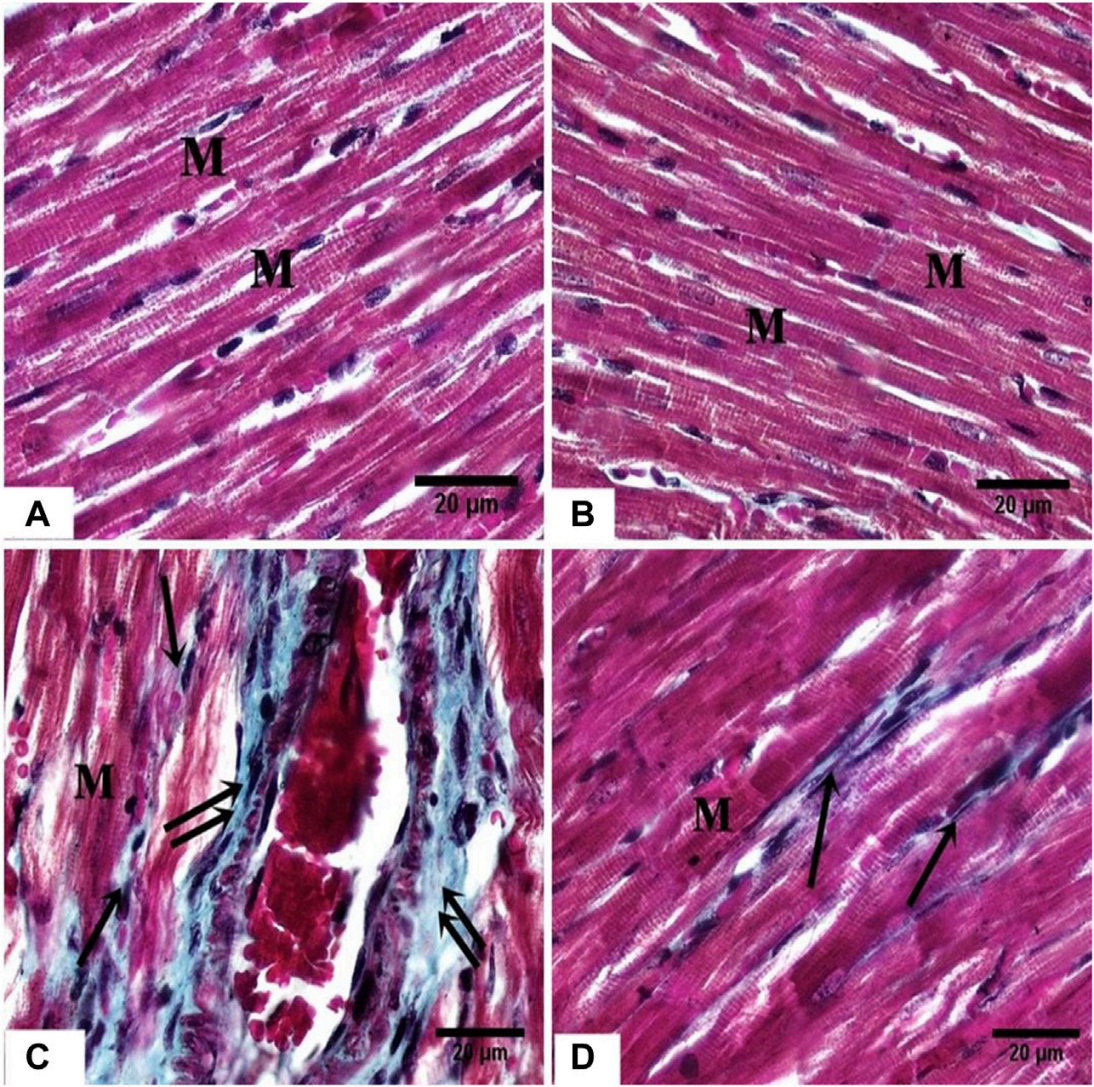
photomicrographs of sections in the rat myocardium **(A, B)** From group (I) and group (II) respectively show the cardiac fibers (M). **(C)** From group III, showing marked deposition of collagen fibers (arrow) between the cardiac fibers (M) and around the blood vessels (double arrow). **(D)** From group (IV) showing mild deposition of collagen fibers (arrow) between the cardiac fibers (M).

In the ISO group, MTC stained sections showed increased collagen fibers between the cardiac myocytes, and in the adventitia of blood vessels (Figure 4C), while in the ISO + DAP treated rats, minimal amounts of collagen fibers were noticed in-between cardiomyocytes (Figure 4D) in comparison with the control (Figure 4A) and DAP (Figure 4B) groups.

#### Immunohistochemically Stained Sections

**FIGURE 5:**
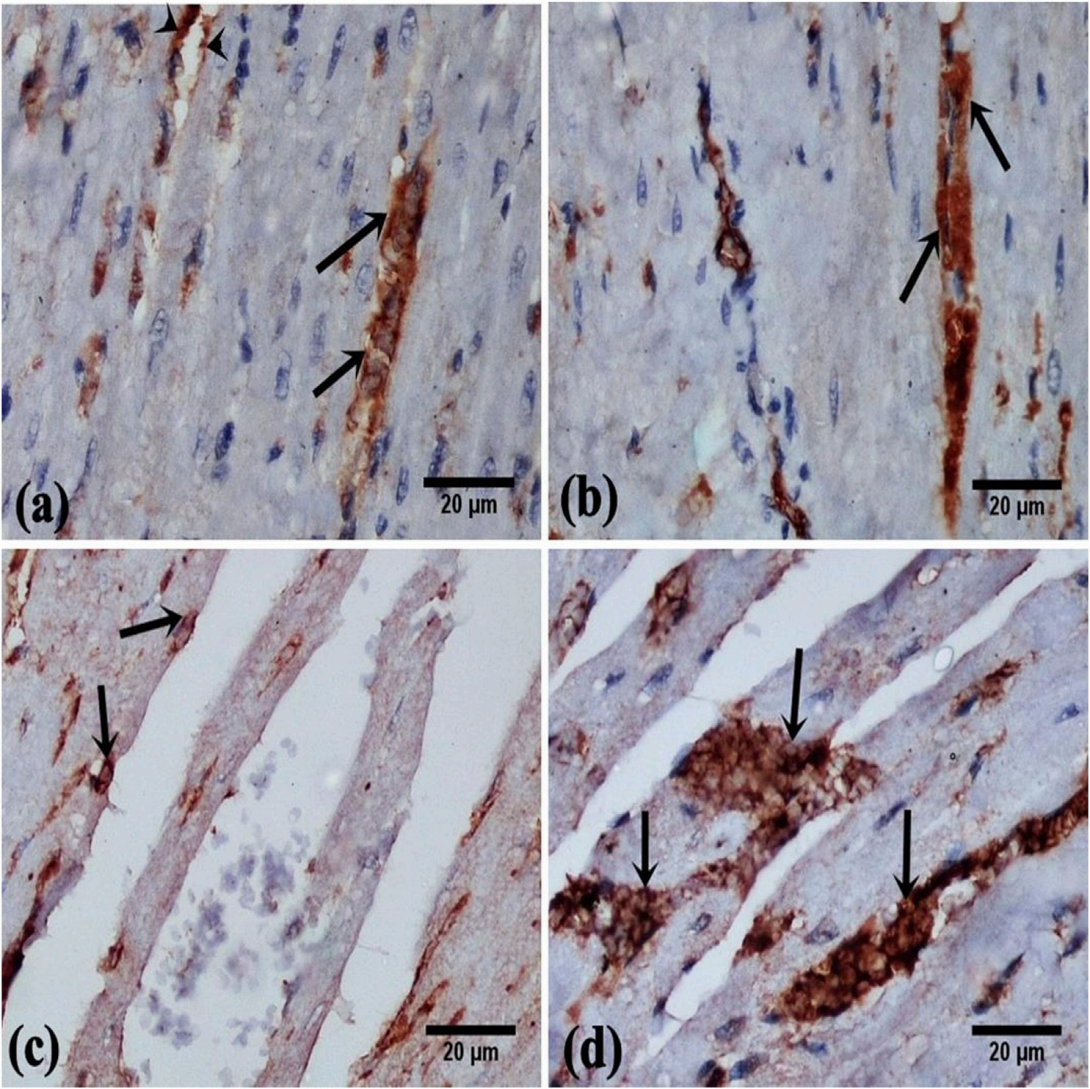
photomicrographs of vimentin cytoplasmic immunoreaction in the rat myocardial sections, in cardiomyocytes (arrow) and in the endothelial cells of the blood vessels (arrow head) **(A, B)** From group (I) and group (II) respectively show moderate immunoreaction. **(C)** From group (III) showing faint immunoreaction. **(D)** From group (IV) showing marked immunoreaction (vimentin immunostaining, X400).

Cardiac sections of control and DAP-treated rodents displayed a moderate immunoreaction for vimentin in the cytoplasm of cardiomyocytes and endothelial cells of the blood vessels (Figures 5A,B). Faint cytoplasmic staining of cardiomyocytes was noticed in the ISO group in comparison with the control indicating a weak immune-expression of vimentin (Figure 5C). Cardiac sections of ISO + Dapsone-treated rats displayed increased cytoplasmic immunoreaction for vimentin in the cardiomyocytes, compared to the previous group (Figure 5D).

#### Morphometric Scores

The assessed mean area % for collagen fibers and mean area % for vimentin immune-expression demonstrated a statistically significant variance between the groups (Table 4). The scores of vimentin immune-expression were less in the ISO group (0.3 fold) than the other ones. Whereas, the score of the mean area percentage of collagen fibers was significantly more elevated (6 folds) in the ISO group than the other ones (*p* < 0.05).

**TABLE 4:** The mean color area percentage of MTC and vimentin staining in rats.

Groups	The mean color area percentage of MTC	The mean color area percentage of vimentin immunostaining
Control	4.63 ± 0.53	4.12 ± 0.52
DAP	4.71 ± 0.61	4.06 ± 0.41
ISO	30.02 ± 1.72^ab^	1.42 ± 0.23^ab^
ISO + DAP	11.62 ± 0.91^abc^	5.41 ± 0.31^c^

Data show the mean ± SEM (*n* = 7).

DAP: dapsone; ISO: isoproterenol; MTC: Massonꞌs trichome stain.

a Significant (*p* < 0.05) variance from the control group.

b Significant (*p* < 0.05) variance from DAP group.

c Significant (*p* < 0.05) variance from ISO group.

**FIGURE 6:**
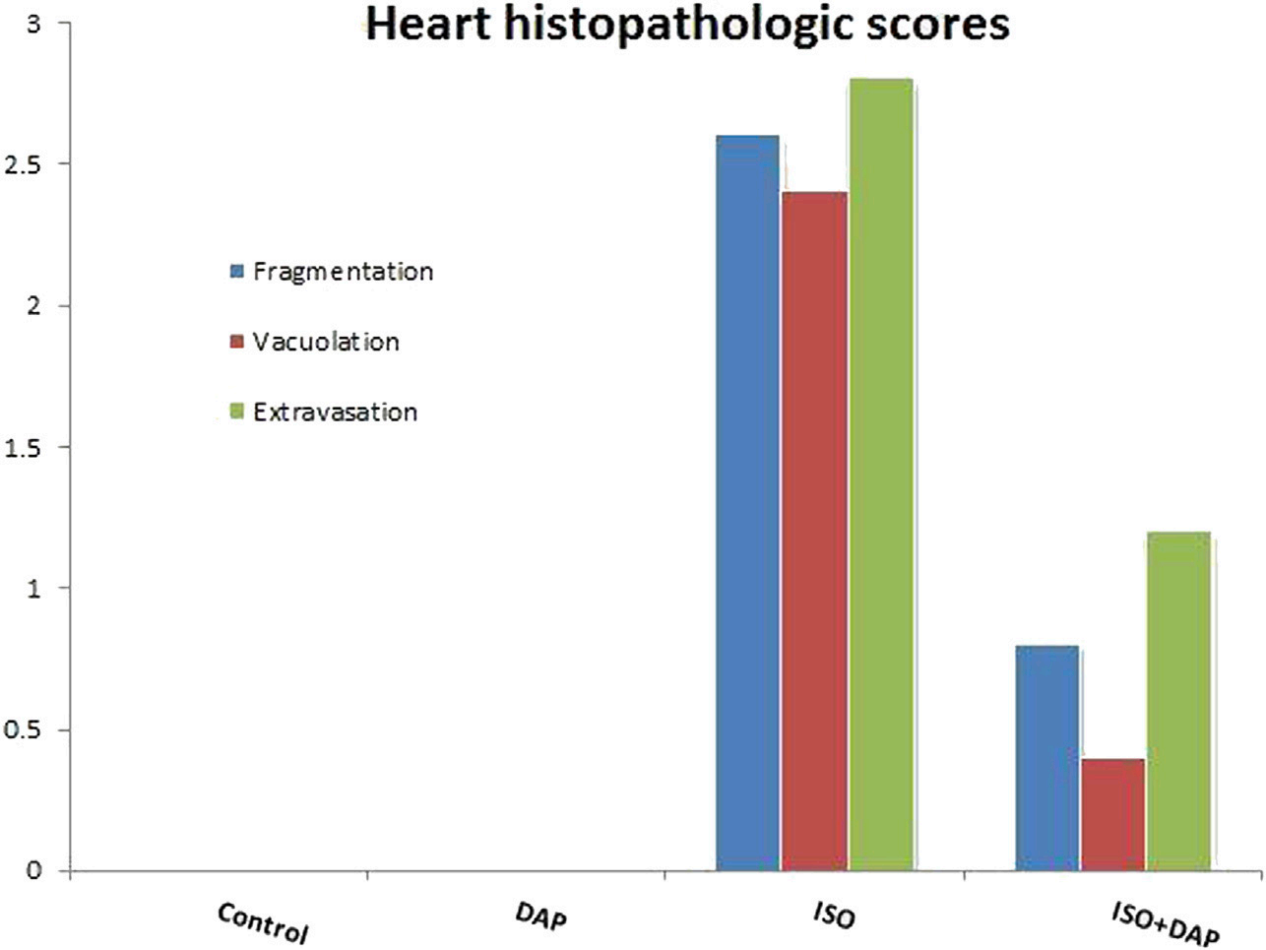
Heart histopathologic scores.

When histological samples were compared, cardiomyocytes fragmentation, vacuolation, and extravasation scores were found to be higher in ISO group compared with the other groups (*p* < 0.05) (Figure 6).

## Discussion

Recently, there is a propitious cardioprotective effect of DAP that is the motivation of this study to curiously examine the mechanisms of its action. The current findings proved the cardioprotective benefits of DAP in isoproterenol-induced myocardial damage in rats.

Although catecholamines are accountable for the regulation of cardiac function, excessive doses lead to ischemic heart disease like myocardial infarction, cardiac hypertrophy, and heart failure. This occurs as a result of abnormal β-adrenergic activation. The abnormalities of cardiac structure and function in rats upon ISO are comparable to human myocardial infarction used to evaluate the possible cardioprotective effect of any novel pharmacological agent (Garg et al., 2016; Khalaf et al., 2020).

ISO is prescribed in low doses for heart block and cardiac arrest. Higher doses are required for hypertrophic cardiomyopathies. The termination of beta-blockers treatment should be in a gradual manner as rapid or sudden terminations precipitate anginal pains. Also, high doses cause desensitization of β-adrenergic receptors preventing overstimulation, decreasing cardiac contractility, and causing an imbalance between oxygen demand and supply of the myocardium. Therefore, irreversible destruction of the myocardium and the presence of infarct-like necrosis take place (Gyongyosi et al., 2019).

The resultant formation of cytotoxic free radicals in the myocardial cells followed by oxidative homeostasis disequilibrium and enhanced lipid peroxidation then mitochondrial damage, inflammatory cytokine production, and intracellular Ca2+ overloading are the commonest factors of the ISO-induced cardiac damage and myocardial cell death (Allawadhi et al., 2018; Sharma et al., 2019). The excessive production of free radicals that are not opposed with enough antioxidants can interact with cellular lipoproteins forming lipid peroxides that cause massive tissue damage (Ibrahim et al., 2018).

ISO-induced oxidative cellular damage was proven in this experiment by the significant elevation in cardiac tissue level of MDA with a subsequent reduction in GSH tissue level.

A sort of cross-talk exists between NO and oxidative stress. The elevation of oxidative stress affected NO primarily through suppressing its synthesis by the uncoupling of enzymes of nitric oxide synthase (NOS) and the inactivation of NO to peroxynitrite with constitutive NOS inhibition (Ibrahim et al., 2018; Khalaf et al., 2020).

Meanwhile, some *in vivo* and *in vitro* researches proved that DAP attenuated oxidative damages. It scavenged antioxidant enzymes e.g., GSH, glutathione peroxidase, and superoxide dismutase as DAP reduced the intracellular free-radical synthesis. Restoration of the balance between ROS and antioxidant enzymes was successfully achieved by DAP (Suda et al., 2005; Cho et al., 2010). Nezamoleslami et al., 2020 reported that DAP had anti-oxidative features by decreasing levels of MDA and myeloperoxidase in the kidney in experimentally induced ischemia/reperfusion injury. DAP decreased the extracellular ROS via the membrane-bound NADPH oxidase enzyme (Wozel and Blasum, 2014).

In the current study, ISO-induced MI showed a significant elevation in cardiac enzymes e.g., CK-MB (an index for acute myocardial destruction), ALT, LDH, and ALK-PH. This was possibly due to the membrane permeability alterations and disintegrity resulting from ISO-induced MI (Khalaf et al., 2020). The cardiomyocyte injury led to the release of enzymes into the blood elevating the serum level of these lysosomal enzymes (Chen et al., 2000). This was supported by the increase in myocardial infarct size, as assessed by TTC staining, was observed in ISO group. The TTC stain reacts with dehydrogenase enzyme in the presence of cofactor NADH to form formazan pigment in viable cells, which are stained as brick red in color. In contrast, infracted cells are having almost lost of dehydrogenase enzyme, and thus, the infracted portion of myocardium remains unstained with TTC staining (Fishbein et al., 1981; Babbar et al.,2013).

This was confirmed by a disturbed histoarchitecture of the myocardium which was noticed in the ISO-injected rodents as distorted cardiomyocytes, vacuolation, edema, pyknosis, and fragmentation. These results were in line with the findings of Helal et al., 2010 and Aziz et al., 2020 that reported the disrupted cardiac muscle fibers with extravasation of red blood cells and accumulation of inflammatory cells between the separated muscle fibers. The degenerative changes associated with isoproterenol (ISO) administration are attributed to the enhanced release of cytotoxic free radicals that stimulated lipid peroxidation and altered the myocardial membrane permeability (Aziz et al., 2020; Khalaf et al., 2020).

On the other hand, co-administration of DAP significantly reduced the cardiac biomarkers and preserved the histological shape and arrangement of myocardial fibers with MI. This was strongly supported by the results of the present study that pretreatment with DAP significantly prevented high myocardial infarct size noted in ISO group. It was proposed that this improvement was due to DAP restoration of oxidative balance and its ameliorative effect on myocardial cells (Sheibani et al., 2020).

Nrf2 is the key player in the oxidative stress pathway (Zhou et al., 2014). Nrf2 regulated the anti-inflammatory reaction and oxidative homeostasis by increasing many anti-oxidant agents and enzymes (Niture et al., 2014; Jones et al., 2015). The current experiment demonstrated a significant supression in Nrf2 level in the ISO group relative to the others. Nrf2 is preserved in the cytoplasm by Kelch-like- ECH-associated protein1 (Keap1) and Cullin3 in normal conditions but with ROS overproduction, Nrf2 migrates into the nucleus destroying critical cysteine residues in Keap1 and interfering with the Keap1-Cul3 ubiquitination system. It initiated the transcription of some anti-oxidative genes e.g., HO-1 and NADPH dehydrogenase (Yamamoto et al., 2008; Howden, 2013). This may explain why HO-1 decreased significantly in the current study with ISO-induced MI.

A large amount of Nrf2 was consumed by excessive oxidative stress. Co-administration of DAP activated Nrf2 signaling pathway to restore the anti-oxidant defenses. The present findings showed that DAP promoted Nrf2 level and arrested its depletion improving the ability to restore the redox homeostasis (Aladaileh et al., 2019). HO-1 down-regulated the inflammatory cytokines and protected the cells against the inflammatory damage (Megías et al., 2009; Zhang et al., 2015). It degraded heme into equimolar amounts of carbon monoxide (CO), free iron, and biliverdin as it is an inducible stress response gene. Inflammation, oxidative stress, NO, and heme stimulates HO-1 (Zhang et al., 2017).

TLRs are present in monocytes and macrophages as they are considered a prevalent type of innate immune response receptors. They can recognize and interact with certain molecular proteins of host apoptosis surfaces. TLR4 is a cornerstone in initiating the inflammatory response via stimulation of Hsp70 in acute injuries as MI and mediating the release of pro-inflammatory cytokines as TNF-α and IL-1β via the stimulation of NF-κB; the nuclear transcription agent (Tao et al., 2016; Ibrahim et al., 2021). This comes in line with the present results of the significant increase of cardiac TLR4 in MI.

Oxidative stress also up-regulated pro-inflammatory cytokine synthesis and this mechanistic link between ROS overproduction and inflammation was demonstrated by (Aladaileh et al., 2019). The release of ROS during MI elevated the levels of the pro-inflammatory mediators and stimulated caspase-mediated apoptosis. This was displayed in the current research by the elevated caspase-3 level in the ISO group. Also, hepatic tissue was affected and showed significant apoptotic changes which appeared in BAX and Bcl-2 markers. The liver has high metabolic activity and perfusion rate. Acute circulatory changes such as cardiogenic shock resulting from an acute myocardial infarction directly influence hepatic blood flow. Lai et al., (2016) supported our results proving the liver injury induced by myocardial injury in rats.

Nrf2 inhibited TNF-α expression indicating that the DAP-induced up-regulation of Nrf2 may target the suppression of ISO-induced inflammatory signaling pathways. DAP is metabolized by the Cytochrome P450 system, specifically isozymes CYP2D6, CYP2B6, CYP3A4, and CYP2C19. DAP metabolites produced by the cytochrome P450 2C19 isozyme are mainly anti-inflammatory (Kwon and Joo, 2018).

Proinflammatory cytokines, including interleukin-6 (IL-6), TNF-α, and IL-1β. In this study, we show that IL-1β has a special role in the modulation of other inflammatory cytokines. Our results show that the increased production of IL-1beta correlates with the cell surface expression of TLR4 (Eskan et al., 2008a). Moreover, blocking the IL-1β receptor greatly reduces the production of "secondary" proinflammatory cytokines such as IL-8 or IL-6. Our data indicate that the induction of IL-1β plays an important role in mediating the release of other proinflammatory cytokines. This was supported by (Eskan et al., 2008b).

An earlier study by Sheibani and his coworkers, 2020 stated that DAP could reduce the pro-inflammatory cytokines mediated cardiotoxicity supporting our results. DAP has a dual effect of anti-inflammatory and antimicrobial functions. It reduced the synthesis of TNF-α and IL-1β by inhibiting the release of the pro-inflammatory messengers (Kwon and Joo, 2018). Another anti-inflammatory mechanism of DAP was the inhibition of neutrophils calcium-dependent functions. Intracellular calcium overload is a critical factor of induction of inflammation (Suda et al., 2005).

In the current experiment, collagen fibers were more concentrated surrounding the blood vessels. This result was in accordance with Salem et al., 2015 who observed the presence of fibrosis in the rat myocardium as a result of the MI. A significant supression in the vimentin immunoreaction was found in MI rats relative to the other groups. This may be attributed to the properties of vimentin; a protein that is essential for the preservation of the cellular structure. Evaluation of vimentin expression revealed cardioprotection against ischemia as vimentin can be used as a therapeutic tool to increase cardiac contractility. (Angoulvant et al.,2004; Merlini et al., 2008). Vimentin is a type III intermediate filament protein that is predominantly expressed in mesenchymal cells (Kidd et al., 2014) and plays a key role in the physiology of the cell, stabilizing the architecture of the cytoplasm, and cellular interactions (Abdel-Wahab et al., 2021).

Morphometrically, a significant elevation in the mean % area of collagen fibers and suppression in the mean % area of vimentin was reported in the MI group in comparison to the other groups. Therefore, MI harmfully disturbed the cardiac morphology and these findings were consistent with earlier studies that reported several MI-induced metabolic and morphological aberrations in the heart tissue (Sudha et al., 2013).

Co-administration of DAP showed a significant decrease in the area % of collagen fibers with a significant increase in the vimentin immune-reaction relative to the MI group. These data were formerly elucidated by the anti-inflammatory mechanisms of DAP in reducing the calcium influx and preventing the calcium-dependent functions of neutrophils (Suda et al., 2005).

## Conclusion

DAP possessed potent ameliorative effects against the ISO-induced MI by inhibiting oxidative stress, inflammatory response, and apoptosis via the Nrf2/HO-1 and TLR4/TNF-α signaling pathways interplay. This can illuminate further studies about DAP use in patients with MI.

## Data Availability

The raw data supporting the conclusions of this article will be made available by the authors, without undue reservation, to any qualified researcher.
